# A review of the postoperative lymphatic leakage

**DOI:** 10.18632/oncotarget.17297

**Published:** 2017-04-20

**Authors:** Shulan Lv, Qing Wang, Wanqiu Zhao, Lu Han, Qi Wang, Nasra Batchu, Qurat Ulain, Junkai Zou, Chao Sun, Jiang Du, Qing Song, Qiling Li

**Affiliations:** ^1^ Department of Obstetrics and Gynecology, First Affiliated Hospital, Xi’an Jiaotong University, Xi’an, Shaanxi, China; ^2^ Northwest Women's and Children's Hospital, Xi’an, Shaanxi, China; ^3^ Cardiovascular Research Institute, Morehouse School of Medicine, Atlanta, GA, USA; ^4^ Center of Big Data and Bioinformatics, First Affiliated Hospital, Xi’an Jiaotong University, Xi’an, Shaanxi, China

**Keywords:** lymphatic leakage, postoperation, complication, chylous ascites, chylothorax

## Abstract

Lymphatic complications are rare, but well-known phenomena, and have been described by many researchers. However, many diagnoses of lymphatic complications are found confusing due to different definition. A literature search in Pubmed was performed for studies postoperative lympatic complications. These complications divided into two parts: lymphatic leakage and lymphatic stasis. This review is about lymphatic leakage, especially, postoperative lymphatic leakage due to the injury of lymphatic channels in surgical procedures. According to polytrophic consequences, many types of postoperative lymphatic leakage have been presented, including lymph ascites, lymphocele, lymphorrhea, lymphatic fistula, chylous ascites, chylothorax, chyloretroperitoneum and chylorrhea. In this review, we focus on the definition, incidence and treatment about most of these forms of lymphatic complications to depict a comprehensive view of postoperative lymphatic leakage. We hold the idea that the method of treatment should be individual and personal according to manifestation and tolerance of patient. Meanwhile, conservative treatment is suitable and should be considered first.

## BACKGROUND

Many surgical procedures may injure lymphatic channels unexpectedly such as lymph node dissections, transplantations and vessels reconstructions, and will lead to iatrogenic lymphatic leakage.

## RESULTS AND DISCUSSION

### Lymphatic circulation

**Figure 1:**
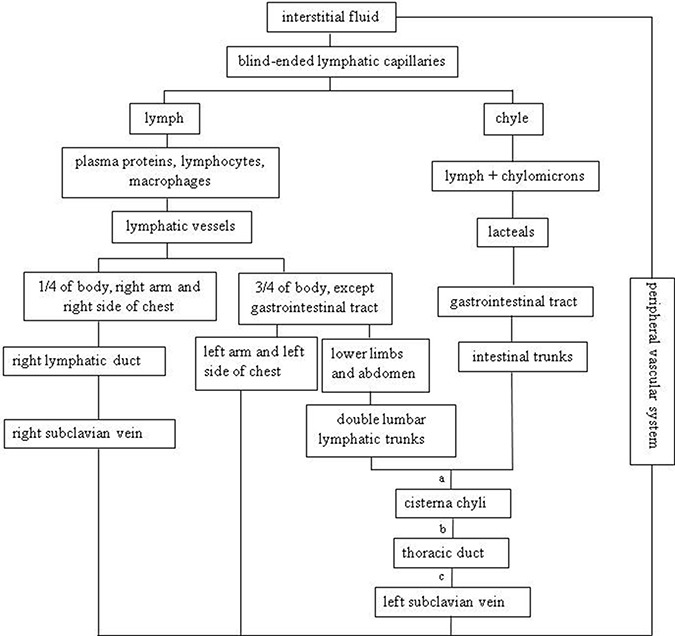
Lymphatic circulation a at the level of the first or second lumbar vertebra; b traverse the aortic hiatus into the right posterior mediastinum; c. at the level of the 4th thoracic vertebra.

Lymphatic circulation can drain proteins and excess interstitial fluid back to the systemic circulation, regulate the immune responses by both cellular and humoral mechanisms, and absorb lipids from the intestine [1]. A schema chart (Figure 1) demonstrates lymphatic circulation clearly. In the physical circumstances, low amounts of fluid, filter into the interstitial tissues continuously, and are collected by blind-ended lymphatic capillaries back into the blood stream. This fluid goes into the cisterna chyli and thoracic duct and ends at the subclavian veins [2].

Cisterna chyli, as a saccular aneurysm, is the termination of the retroperitoneal lymphatic pathways and the beginning of the thoracic duct. According to the anatomic datum, half of the cases don't have cisterna chyli, as it is replaced by a variable lymphatic plexus [3]. For thoracic duct, it exists in paired form, right and left thoracic ducts during the development of the embryo, but the only certain portion will develop into an adult thoracic duct. So in some cases, thoracic duct circulates into right and left branches. Right branch joins the right subclavian or internal jugular vein [2]. A radiological study shows that the incidence of a visible, right-sided duct is 4% [4]. Another appearance type is the single right-sided thoracic duct emptying into the right internal jugular vein through three separate terminal branches [5].

Fatty acids less than 10 carbon atoms will be transported into the portal venous system directly, while fatty acids greater than 10 carbon atoms will be absorbed by lacteals and lymphatic capillaries of small intestine, forming chylomicrons [6, 7]. The mixture of lymph and chylomicrons is called chyle [8] which is milky white tint [9], odorless [10] and strongly bacteriostatic due to the large number of lymphocytes. It is estimated that 3 to 5 Liter per day or 60 to 190 ml per hour of lymph fluid passes through the thoracic duct during the absorption phase of digestion [6]. 50% to 90% of lymph fluid in cisterna chyli and thoracic duct comes from intestines and liver. Fasting before operation may decrease lymphatic flow less than 1 ml/min dramatically, while it may increase to more than 200 ml/min after a normal diet recovers [11, 12]. The amount of the lymphatic flow may increase and the size of lymphatic ducts may be magnified under the following circumstances of hypertension and cardiac diseases, particularly mitral valve dysfunction and left ventricular aneurysm [3].

### Definition of lymphatic channel leakage

**Figure 2:**
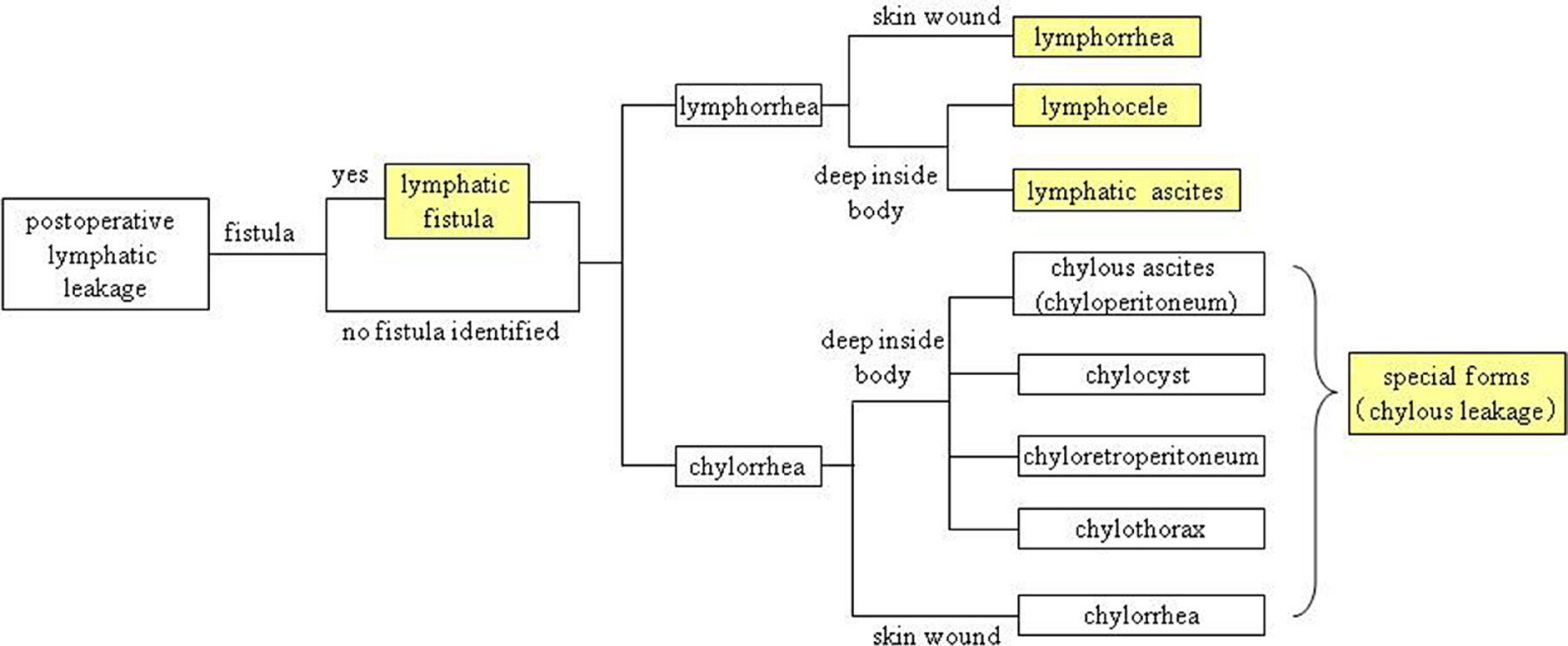
Classification of postoperative lymphatic leakage

**Figure 3:**
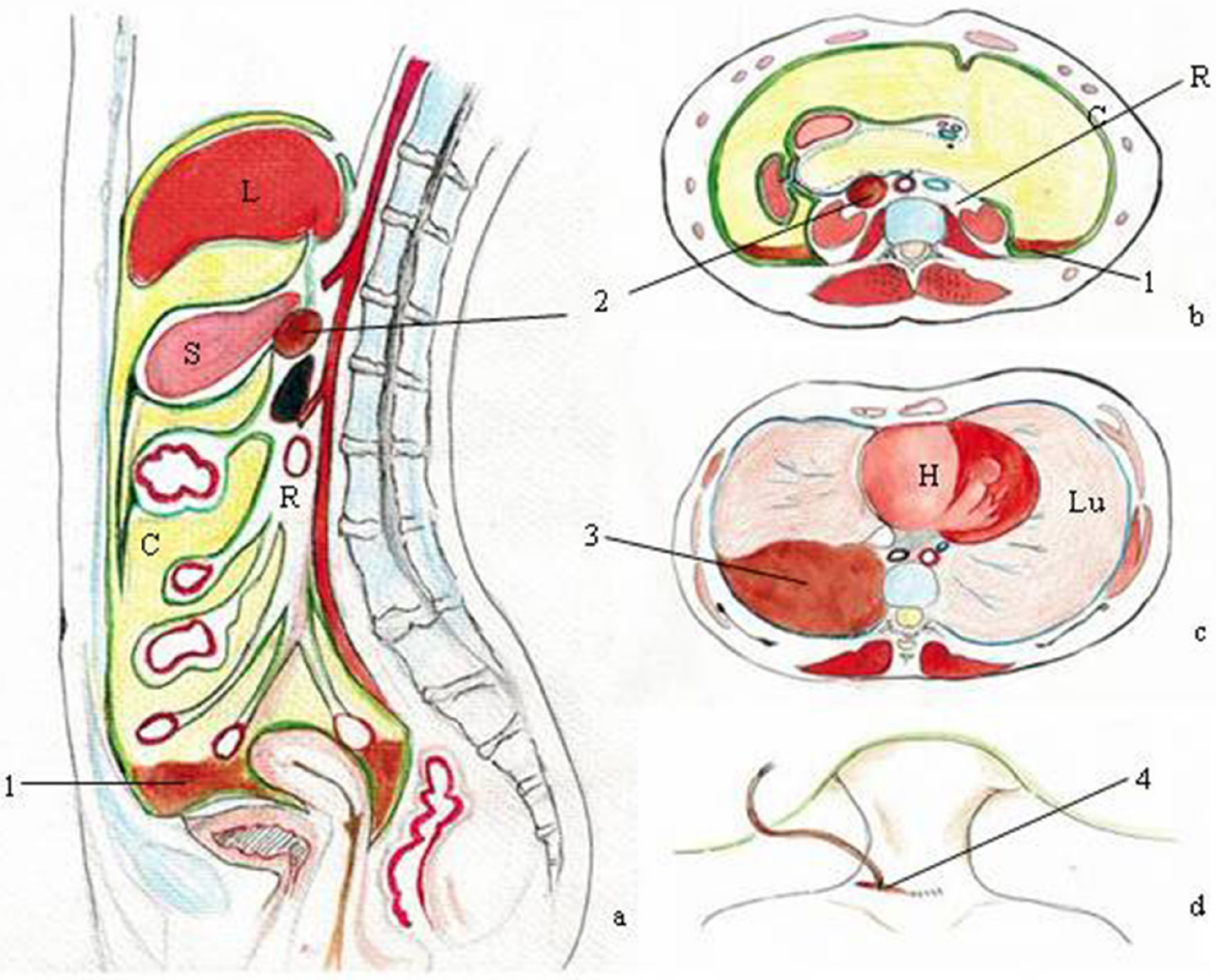
Pictured here is the postoperative lymphatic leakage (a and b) peritoneum anatomy (cross section and longitudinal section); c: thorax anatomy; d: sketch of cervical incision; L: liver; S: stomach; C: peritoneal cavity; R: retroperitoneal space; H: heart; Lu: lung; 1: the lymphatic ascites or chylous ascites; 2:lymphocele (it can also exist in peritoneal cavity or in other soft tissue not only retroperitoneal space); 3:the chylothorax; 4: the lymphorrhea or the chylorrhea; Lymphatic fistula and chyloretroperitoneum are not showed in this picture.

During operation, the trauma of lymphatic system results in the postoperative leakage of lymphatic fluid. Many kinds of postoperative lymphatic leakage have been reported, which includes lymphatic ascites (lymph ascites) [13–15], lymphocele [16, 17], lymphorrhea [7, 18], lymphatic fistula [19–22], and some special forms of lymphatic leakage: chylous ascites (chyloperitoneum) [23–26], chylorrhea [27–29], chyloretroperitoneum [30, 31], chylothorax [32, 33]. In order to describe them readily, we categorize them into five types owing to different consequences and characteristics (Figure 2 and Figure 3).

### Lymphatic ascites (Lymph ascites)

Lymphatic ascites is the accumulation of straw-colored or clear lymph fluid in the peritoneal cavity with a similar composition of creatinine and blood urea nitrogen (BUN) to their serum [13]. Biochemical tests show a low level of triglyceride even after a high fat diet [14, 15]. Most of lymphadenectomies lead to the leakage of lymphatic vessel, but they usually stop spontaneously without the consequence of symptomatic ascites. About 9 of 110 patients (8.2%) were identified with lymphatic ascites by routine weekly sonograms after surgeries. However, only 5 of these 9 patients were symptomatic and required interventions eventually [8]. Since pelvic and para-aortic lymphadenectomy being performed during genito-urinary malignancies procedures [34], lymphatic ascites with the low incidence of 2.7% to 4.5% [8, 34] seemingly have a special favorite in surgeries of gynecologic cancers such as endometrial and cervical cancers [15] (Supplementary Table 1 and Supplementary Table 2). There is another lymphatic ascites after appendectomy reported [14], which is really rare.

### Lymphocele (lymphocyst)

Lymphocele, same as lymphocyst [16, 17], is a cystic filled by clear lymph fluid with no inflammatory or granulomatous reaction at the leakage site. There may develop a swelling pocket cavity under healing wound in soft tissue with or without septa [35–37]. Lymphocele often occurs within 3 ∼ 8 weeks or 1 year occasionally after surgeries [38]. Because of self-limiting, most of postoperative lymphocele is usually asymptomatic, undiagnosed and self-healing without any treatments [38, 39]. Only 4% – 7% of postoperative lymphocele is symptomatic [16] due to self-absorption disorder. The mean diameter of a symptomatic lymphocele is usually more than 5 cm [16]. And it will require some interventions [38] when pain, infection, lymphorrhea on the fresh wound or compression of vital structures occurs [17].

Lymphocele occurs in many surgeries on various parts of body, including pelvis [40], mediastinum, axilla, neck, aorta and peripheral vasculature [41]. Pelvic lymphocele is usually related to pelvic lymphadenectomy and renal transplantation [17]. 0.6% to 22% of kidney transplantations at the lower or upper medial region develop postoperative lymphoceles [38, 42]. Inguinal lymphocele is also a well-known complication of inguinal lymph nodes dissection (ILND) for penile and vulvar cancer, with an incidence ranging from 5% to 87% [43, 44].

As the influence factors, body mass index (BMI) and the number of resected pelvic lymph nodes reach the statistical significance to lymphocele incidence [38, 45]. Gery et al. [37] observed 163 patients with laparoscopic extra-peritoneal para-aortic lymphadenectomies. As for the lymphocele incidence, there is no significant difference found (*p* = 0.80) whether using ultrasonic advanced energy instruments or not. The incidence of lymphocele is lower without postoperative radiotherapy (*p* = 0.01), but no significant difference shows between pre/postoperative chemotherapies (*p* = 0.10) [38]. Symptomatic lymphoceles are more frequent after open surgeries compared with laparoscopies [16]. The risk of lymphocele is also higher in lower lumbar surgeries because of a higher density of lymphatic channels existence [46].

### Lymphorrhea

Lymphorrhea was once used as replacement of lymph ascites, chylous ascites, chylothorax or the lymph fluid exudation, which really confused us [16, 47]. Actually lymphorrhea is lymphatic exudation on the wound after slight trauma of lymphatic vessels. Lymphorrhea after the trauma, deep inside body, will heal itself in most of the situations or form into lymph ascites, lymphocele [7, 18] occasionally. Ilzecki et al. describe lymphorrhea as light color fluid profusely soaked dressing [48]. Ghezzi F et al. [16] ever defined lymphorrhea as the ultrasonographic finding of free lymphatic fluid in the peritoneum. This is not accurate since we cannot find the difference between lymph ascites or chylous ascites. Lymphorrhea may occur after the vascular reconstructive surgeries, such as a reconstructive procedure of the superficial femoral artery [48]. And 10% – 16% of cases after vein graft harvesting for infrainguinal arterial bypass suffer from lymphorrhea [13].

### Lymphatic fistula

Lymphatic fistula includes lymphocutaneous fistula [49–51] and lymphoperitoneal fistula [52, 53]. Lymphocutaneous fistula prompts the formation of lymphorrhea, chylorrhea and other associated lymphatic leakage. Lymphoperitoneal fistula is one of reasons of lymphatic ascites and lymphocele. There should be an objective existence of fistula identified by lymphangiography or lymphoscintigraphy. Meanwhile, a pilot study and a subsequent clinical trial show that the lymphatic fistula form after 48 h of continuing leakage [54]. Lymphatic fistula can form due to the increased pressure in lymph vessels or the reflux of capillary lymph vessels from the lymphatic channel leakage, which can dilate the capillary lymph vessels and destroy the normal structure of skin or tissue [50].

Lymphatic fistula is common after inguinal or axillary lymph nodes dissection in different urologic, gynecologic, dermatologic cancers [55] and arterial reconstruction [51]. The incidence of lymphatic fistula in thyroid surgery is 0.5% to 2.5%. And it will be a little much higher with lymph nodes dissection for thyroid cancer [22].

Besides the types of postoperative lymphatic leakage mentioned above, there are some special forms of lymphatic leakage such as chylous ascites (chyloperitoneum), chyloretroperitoneum, chylothorax and chylorrhea. This classification depends on the following reasons: ① Lymph fluid is clear or straw-colored ascites similar to the serum of patients [13]. Chyle, the mixture of lymph fluid and chylomicrons [8], is milky white tint [9], odorless [10] and rich in triglycerides. ② The lymph fluid and chyle distribute to different lymphatic channel vessels [8], that means they may reminder doctors of the different preliminary locations of the lymphatic channel leakages. ③ Since rich content of triglycerides, the loss of chyle is more likely to induce the nutritional deficiency, immunologic dysfunction or some other complications [34]. ④ The treatments using medium-chain triglyceride diet, somatostatin analogue and so on are thought to be more effective to chylous leakage than lymph leakage [34, 56]. ⑤ Compared with postoperative lymph fluid exudation, the drainage of milky white chyle will increase the fear, anxiety and other unhealthy emotion of patients and reduce the trust in their doctors according to clinical observation. All in all, the special types of postoperative lymphatic leakage are different to the other types of lymphatic leakage.

### Chylous ascites (chyloperitoneum)

Postoperative chylous ascites, or chyloperitoneum, is the pathologic accumulation of chyle in the peritoneal cavity [52, 57–62]. The leaking fluid is rich in triglycerides (>200 mg/dl) [23–26], odorless, alkaline and sterile. The symptoms of chylous ascites usually occur at average 4.1 days after surgery due to the dietary intake at post-operation day (POD) 2. It usually forms as a result of surgical trauma of the thoracic duct, cisterna chyli or their major tributaries [34, 59].

There are three mechanisms in the formation of chyloperitoneum, including ① direct leakage of chyle through a lymphoperitoneal fistula associated with abnormal retroperitoneal lymphatic vessel; ② exudation of chyle through the walls of the retroperitoneal lymphatic without a visible fistula; ③ exudation or leakage of chyle after the rupture of dilated lymphatic of the bowel wall and mesentery caused by obstruction of the lymphatic vessel at the base of the mesentery, cisterna chyli, or thoracic duct [52].

Different incidences of postoperative chylous ascites have been analyzed in many surgeries including post-chemotherapy laparoscopic retroperitoneal lymph nodes dissection testicular cancer (23 of 329 patients, 7%) [63], or for stage IIB nonseminomatous testicular cancer (5 of 24 patients, 20.8%) [64] high incidence probably due to lymphatic metastases; surgery for abdominal neuroblastoma (32 of 160 patients, 20%) [65]; surgery for colorectal cancer (48 of 779 patients, 6%) [66]; retroperitoneal lymphadenectomy (24 of 1258 patients, 2%) [58]; laparoscopic donor nephrectomy (10 of 4636 patients, 2%), open donor nephrectomy (2 of 3047 patients, 0.6%) [61], laparoscopic nephrectomies (9 of 1159 patients, 0.77%) [67]; gynecological surgery with pelvic or/and para-aortic lymph node dissection (7 of 4119 patients, 0.17 %) [23]; and pancreatic resection (49 of 1921 patients 2.6%) [24].

Right colectomy has a significantly higher incidence rate of chyle leakage (9.6%) than left colectomy (2.6%) and anterior resection (2.8%). The reason may be the abundant lymphatic tributaries posterior to the abdominal aorta are close proximity to the cisterna chyli [24]. Resection of inflammatory aortic aneurysm, especially emergency surgery for a ruptured abdominal aortic aneurysm has a particularly high risk of chylous complications for the extensive and difficult dissection during the surgery. What's more, abdominal aortic surgery is said to be the most common etiology of postoperative chylous ascites, and causes 81% of all chylous complications due to the surgical injury of the retroperitoneal lymphatic glands or cisterna chyli [59]. Lymphatic metastases, number of lymph nodes removed and damage of lymphatic channels may increase the incidence of lymphatic leakage [64, 65]. This view needs more supports of clinical data. Open donor nephrectomy carries a lower incidence of chylous ascites than laparoscopic donor nephrectomy. However the reason is unclear, maybe for the easily suture ligation or clip [61]. Laparoscopic procedures seem to have a higher risk of unrecognized lymphatic injury. And the author suggests surgeons to coagulate the lymphatic channels adequately, even if there is no lymphatic leakage found [31, 61]. However, some other people reported a lower incidence of chylous ascites with laparoscopic than laparotomy surgeries [16, 68]. Many relevant factors have been reported to chylous ascites, such as older age, female sex, preoperative chemotherapy, concomitant vascular resection, and low preoperative albumin, tumors fed by the superior mesenteric artery, increased intraoperative blood loss, and early enteral feeding, and we need detailed big clinical data to analyze the high risk factors [69].

### Chyloretroperitoneum

Chyloretroperitoneum is the chyle accumulating in a restricted limited space, retroperitoneal lacuna and not in the peritoneal cavity as chylous ascites [30]. There are few articles in PubMed not only because of the rare incidence [31], but also for the misdiagnosis as chylous ascites.

Chyloretroperitoneum has a special interest in urological surgeries [59], such as retroperitoneal lymph nodes dissection (RPLND) for testicular cancer, radical nephrectomy, donor nephrectomy and so on [31]. Anterior spinal surgery with or without diaphragm splitting is another relevant surgery to chyloretroperitoneum. The surgery below L1 level without diaphragm splitting is prone to injure the cisterna chyli or their tributaries [12]. Chylothorax may occur secondary to postoperative chyloretroperitoneum [12, 70].

### Chylothorax

Postoperative chylothorax is the accumulation of chyle in the pleural space due to the leakage of the thoracic duct or its branches. It is a rare but well-known lymphatic complication during the thoracic procedures [32].

Chylothorax occurs after many surgeries such as esophagectomy [25, 71, 72], pediatric congenital heart surgery [9, 73, 74] and pulmonary procedure [75, 76]. It can be detected within 3 days due to the beginning of oral intake food on POD 1 in most patients. But a rare late-onset chylothorax occurs 40 days after a right middle lobectomy [32].

Different surgery procedures usually have different incidences of chylothorax, such as esophagectomy 2.1% [25] − 2.5% [77], thoracoscopic esophagectomy 1.1% [78], pediatric congenital heart surgery (0.6% to 2%) [9], and pulmonary resection (1.4% to 4%) [26, 32, 79]. Miao et al. [77] reported a high BMI associated with decreased incidence of chylothorax following esophagectomy. What is more, the incidence of postoperative chylothorax of left-sided mediastinal lymph node dissection (MLND) is less than that on the right side [32]. And patients with pathologic N2 disease, which was defined as pathologically confirmed metastatic cancer in an N2 lymph nodes, had a higher incidence rate after robotic resection [26].

### Chylorrhea

Chylorrhea is a special form of lymphatic leakage [27, 29]. Compared with lymphorrhea, the only difference is the chyle exudation not lymph fluid. There are very few articles regarding chylorrhea due to confused classification with other types of lymphatic leakage. This is mixed up with lymphorrhea and chylous ascites, usually.

### Lymph leakage and chylous leakage

Even when we have defined most of the kinds of postoperative lymphatic leakage, there are still some types which can't be classified. For example, the lymphatic fluid drains from the drainage tube after the thyroid surgery with no lymphocele or lymphorrhea found. As one kind of lymphatic leakage, it can also be called chylous leakage with chyle fluid drainage or lymph leakage with only lymph fluid [10, 31, 80, 81].

Laparoscopic procedures seem to have a higher risk of unrecognized lymphatic injury since lymphatic leakage might be overlooked by CO_2_ pressure [31]. Monopolar, bipolar or ultrasonic coagulation in laparoscopic surgery will destroy the lymphatic tracts [10]. 23 of 381 (0.6%) patients with malignant pancreatic tumors undergoing pancreaticoduodenectomy get the chylous leakage at POD 1 – 2 days after taking food. And the incidence is unrelated to gender, age and tumor pathological pattern but significantly associated with lymphatic metastasis [82].

### Complications of lymphatic leakage

Loss of fluid, triglyceride, lymphocyte and immunoglobulin [24, 50, 66], from the leakage of lymphatic vessels may lead to dehydration, nutritional deficiency [57, 61] and immunologic dysfunction [9, 59]. Pain and prolonged hospital stay (about 12.4 to 20.4 days) are the most common influence of lymphatic leakage [49]. At the same time, it will also will increase the infection rate [22, 38, 54] even with the negative microbiological test. Compression of vital structures may often happen in chylous ascites, lymphocele and chylothorax [22]. The others are less mentioned or with no clinical symptom, such as dehydration, nutritional deficiency, hypoalbuminemia, immunologic dysfunction or pyemia [22, 83]. Theoretically, all of these complications can occur for a long time and a large loss of lymphatic fluid. A rare complication of postoperative chylous ascites is a skin rash which rapidly progresses to widespread itchy erythema. And it almost regresses after a pigtail drainage tube is placed and approximately 3.5 Liter of odorless yellow-white fluid drained off [10].

### Diagnosis

Clinical symptoms include abdominal fullness, dyspnea, lasting pain, nausea and vomiting, malnutrition and hypoproteinemia [84]. Before diagnosis of lymphatic leakage, we need first rule out of other post-operation complications, such as malignant ascites, hemoperitoneum, urine ascites due to injury of bladder, malignant pleural effusion, inflammatory exudation in body cavities, purulent exudate and so forth [68]. Collection and biochemical analysis of drainage is necessary. Oral intake related drainage increasing should catch your attention of lymphatic leakage. Physical signs of lymphatic leakage contain the clear or milky fluid exudate on the wound, swelling under the wound or ascites [12]. The adjuvant examinations include oral contrast test, computerized tomography (CT), lymphangiography, lymphoscintigraphy, laboratory examination and diagnosis by paracentesis. All this can be chosen to make an exact diagnosis.

### Oral contrast test (a fat-containing test meal)

Lymphatic flow will be less than 1 ml/min during pre-operative fasting time, but it may return to more than 200 ml/min after normal diet post-operation [11, 12]. Increasing rapidly from fasting to oral intake and/or turning clear to milky drainage fluid are the evidences of lymphatic leakage, especially the leakage of gastrointestinal tract [14]. High fat diet, an extensive amount of double cream [25, 85], is used to detect the leakage of lymphatic channels [38, 86]. That can be called “chylous lymphangiographic effect” [86]. However, another study holds the view that high fat diet only shows the diffusion of lymph leakage [57], but it's difficult to exactly point out the leakage. Sudan black in a concentrated fatty meal can facilitate the identification of leakage sites [57]. Diet contrasts combining with imaging studies are also used to locate the site of leakage such as ^13^Carbon-palmitic acid and ^123^Iodine pentadecanoic acid [59].

### Diagnostic paracentesis (US/CT/MR-guided) and laboratory examination

CT, ultrasonography (US) or magnetic resonance(MR) [53, 87] used in the diagnosis of lymphatic leakage, especially lymphocele and chylous ascites [57] can show us the hydrops [88], the cyst of body cavity [31] and location the leakage [89] particularly when there is suspicion of thoracic duct injury [85]. US- or CT- or MR- guided paracentesis and following by laboratory examination are widely used in diagnosis. Lab analysis findings include ① milky white or clear color, odorless, alkaline sterile fluid [9]; ②triglyceride-rich [90] (above 200 mg/dl [38, 91] or 2 to 8-fold of plasma [59, 61]); ③ high lymphocytes and proteins [9, 91]; ④ small amounts of cholesterols [12, 58, 91] and so on.

### Lymphangiography

Injection of ethiodized oil [59, 92, 93], patent blue V dye [94], isosulfan blue [95], indocyanine green (ICG) [13, 50] into lymphatic vessels on the dorsum of the foot can make a real-time visualization of the lymph flow and identify the broken lymphatic vessels under direct vision. Bipedal lymphangiography can both be used in diagnosis and therapy to close the leakage [14, 96]. The reason may be that the inflammatory reaction caused by the contrast leads to the fibrosis and obliteration of leakage. Lymphangiography with thoracic duct embolization is also a well-described technique of refractory thoracic lymphatic leaks for diagnosis and treatment [97].

Several complications have been described of lymphangiography, including the tissue necrosis, fat embolism and hypersensitivity relating to the volume and type of contrast agents [85]. However, since it is difficult to treat at the meantime even on direct vision of lipiodol leakage, lymphangiography is not appropriate in dealing with lymphocele [36]. What's more, other side effects such as painful, tedious, poor reproducibility and some other negative results, increase the limitation of extensive use of lymphangiography in some fields [61].

### Lymphoscintigraphy

Lymphoscintigraphy was first introduced using ^198^Au-colloid in 1953, which was then replaced by ^99m^Technetium-colloid because of its high absorbed radiation at the injection site [98]. This technique is a nuclear medicine examination whereby a radiotracer is injected into the lymphatic system via the feet commonly. And then the images are obtained to monitor the flow of lymph to assess the rate of tracer transport, the number, size, distribution of lymph and identify any defects in the lymphatic vessels [8]. Lymphoscintigraphy can also show the collateral, fistula or lymph reflux [98] or reveal abnormal lymphatic stasis at the diaphragmatic level [8, 57]. And it is helpful in showing the lymphatic way that fills and maintains the lymphocele [44]. However, it's the same as lymphangiography, no obvious lymphatic leakage point revealed by lymphoscintigraphy was also reported [13].

### Treatment

Lymphatic leakage is a rare postoperative complication with controversial therapeutic methods. Many scholars devote themselves to finding the best way of treatment. However, so far, there is still no treatment guideline [58]. The following views are prone to convince us the basic principles of treatment. Firstly, before the exact method is taken, an assessment of an individual patient should be performed on consideration of prolonged hospital stay, patient's tolerance, compliance to trying fasting protocols, evidence of impairing health status or wound infection and so on. Secondly, treatments should be based on a step-up approach from conservative treatment for several weeks up to 2 months to surgical intervention [38, 58]. 66% to 77% of patients can be successfully treated by conservative methods [9, 22, 59, 63]. Two articles describe a successful conservative treatment to all of their 48 and 23 patients [24, 82], respectively. Shao et al. also emphasized that lymphatic leakage is a self-limiting complication, which may heal within 2–3 weeks without further intervention. And lymphatic fluid can be absorbed by the peritoneum [99]. Conservative therapy including ① diet control (high protein, low fat, medium-chain triglyceride diet), the fasting and total parenteral nutrition; ② application of drugs (somatostatin analogue, vasoconstrictor pancreatic lipase inhibitor, diuretics, traditional Chinese herb medicine [6, 83]); ③ paracentesis and sclerotherapy [22], pressure dressing.

On the contrary, some authors think surgical intervention should be performed first for lymphatic leakage, since early ligation or suture of the leakage site is helpful to avoid metabolic complications and shorten hospitalization days [100]. If drainage volume is greater than 1000 − 1500 ml/day for more than 5 days during conservative treatment, surgical interventions should also be considered [12, 82]. The refractory leakage prompts a more aggressive approach as surgical repair including: ① peritoneovenous shunt; ② direct lymphostasis by suture ligation of the disrupted lymphatic channel; ③ surgery combining with fibrin glue [59].

### Medium-chain triglyceride (MCT)

MCT should be considered first when treating the lymphatic leakage. 50% to 90% lymph fluid of cisterna chyli and thoracic duct come from the intestine and liver [11]. Long-chain fatty acids will undergo a second esterification and then enter the lymphatic system as chylomicrons, while medium-chain fatty acids pass directly into the portal system and couple to albumin [9]. Low fat elementary diets are absorbed directly into the portal venous system and bypass the lymph vessels [6, 8, 101]. These treatments lead an especially MCT diet for decreasing the bowel absorption of fat. It is the basic theory of lymphatic leakage treatment [38, 62, 83, 89]. Leibovitch et al. suggest us that MCT diet should be tried before total parenteral nutrition [59]. Some people hold the view that a medium-chain triglyceride diet is more efficacious to chylous leakage than to lymph leakage due to the high cholesterol content in chylous fluid [34]. However, Frey et al. show the improved lymphatic ascites after using MCT [8].

### Total parenteral nutrition (TPN)

TPN has been applied in most of the cases to correct the nutritional consuming and offset metabolic impairment. TPN allows bowel rest and decreases the production of lymph. In the same time, it supplies proper proteins, vitamins and electrolytes to promote protein synthesis, enhance plasma colloid osmotic pressure, and increase peritoneal effusion absorption [82]. It can be used alone or combined with other treatments [38]. The combination of TPN with somatostatin shows a significant effect in lymphatic leakage [61, 102]. Pabst et al. suggested that total parenteral nutrition should be applied immediately after the diagnosis of chylous ascites [59]. As for side-effect, TPN is not always effective [102] and it may bring several complications such as infection, thrombosis, cholestasis or mucosal integrity disorder with a very low incidence [9]. Seow et al. [6] described a patient who developed a parenteral nutrition line sepsis. However, Milonakis et al. [9] found no case meeting such problems in their 18 patients with chylothorax. What's more, after 2 – 6 weeks of TPN, 60% – 100% of cases are resolved.

### Somatostatin

Since the availability of somatostatin was emphasized in dealing with post-operative lymphorrhea in 1990 [31], it has been rapidly used in many cases. Somatostatin, a peptide hormone consisting of 14 to 28 amino acids, is highly effective in patients with high output lymphatic leakage [5, 58]. The mechanism is not clear. Some researchers think that it may reduce lymphatic production and concentrate the lymphatic vessels directly [58]. The receptors of somatostatin may distribute in various regions, including the pancreas, vascular tissue and gastrointestinal tract [18, 31, 58], even the smooth muscle cells of thoracic duct [58]. Therefore, it is speculated that somatostatin might induce the contraction of smooth muscle cells in blood vessels [58], and inhibit secretion and absorption of gastric, pancreatic and intestinal systems [9, 58]. And it may decrease lymphatic and splanchnic blood flow and decrease the hepatic venous pressure gradient. There might also be a direct action on lymphatic vessels [18]. Somatostatin is administered to patients with no response to the primary therapy such as MCT or TPN.

Octreotide, as one of the somatostatin analog, has a much longer half-life. It is widely used in the treatment of lymphatic leakage and has shown to be highly effective [70] in controlling lymphatic flow [5, 58]. A typically drastic decreasing in the output of the lymphatic leakage [102] occurs after 24 to 72 hours with the application of octreotide [58, 59, 103].

Two retrospective studies shown a success rate of 87% – 90% in the use of octreotide as an adjunct to conservative managements for chylothorax [31]. On the contrary, some authors doubt the effectiveness of somatostatin [61], since only two of six patients showed response to somatostatin [9].

### Negative pressure wound therapy (NPWT)

Lymphorrhea after vascular surgery, such as femoral endarterectomy, aortobifemoral bypass, inguinal lymph nodes dissection need vacuum-sealing drainage (NPWT) [38, 104]. According to an animal test, NPWT can increase the granulation tissue in the wound by 103.4% ± 35.3% [35]. NPWT is supposed to promote wound healing by increasing blood flow, removing the wound inhibiting factors and decreasing the bacterial count [35]. Many authors describe the successful use of NPWT for 11 to 19 days after the femoral arteriotomy and inguinal lymph nodes dissection [105, 106]. On the contrary, some authors declare that it is unnecessary for the routine use of vacuum drainage in groin wounds [54]. And vacuum drainage is uncomfortable with no distinct advantage in preventing its complications.

### Drainage and sclerosing agents

Drainage can relieve symptoms by decreasing the accumulation of lymphatic fluid. It is frequently applied in most of lymphatic complications, such as lymph ascites, lymphocele, chylous ascites, and chylothorax [8, 16, 23, 26]. The volume of drainage is also an important index to evaluate the condition and to make the next therapeutic protocol of the patient. Percutaneous drainage is a safe method to deal with lymphatic leakage, especially lymphocele but with a high recurrence rate (up to 50%) [17, 43]. Combining with percutaneous drainage with a sclerosing agent, including doxycycline, tetracycline, bleomycin, OK-432, povidone-iodine, lipiodol and ethanol, can significantly decrease the recurrence rate [78, 96, 107]. The success rate of sclerosing treatment in the literature is 88% – 100% [60, 88]. Their standard of success is ① the recovery of clinical signs and symptoms; ② the maximal drainage less than 10 ml/day; ③ imaging evidence of lymphocele disappearance [88]. Pleurodesis dealing with the chylothorax by injection a mixture of 10 Klinische Einheit unit of OK-432 through a chest tube is effective for 13/15 of patients who have been treated by conservative treatment for 3 days with more than 300 ml/day drainage [76].

Another mentioned conservative treatment is traditional Chinese herb medicine (TCHM) reported by Xiu et al. [83]. It is an effective way to cure lymphatic leakage, with a marked decrease for 1 day after the use of TCHM to all their 6 patients. And their patients completely recover in 4 to 8 days. But we need more data to support the opinion on the usefulness of TCHM.

In short, after appropriate treatment most of the postoperative lymphatic leakage can be cured [61].

### Surgery

Peritoneovenous shunt [100] and operation under direct vision with or without the use of fibrin glue [91] is the most common surgical treatments to lymphatic leakage. Most of operations are taken following the failure of conservative treatment [60]. The surgery is particularly recommended when ① leakage sustains for more than 2 weeks; ② drainage volume reaches more than 1 Liter, even after 1 week; ③ the patient starts to experience metabolic complications [83].

Lymphangiography and concentration of high fat diet intake are often used to locate the leakage. Once it is detected, ligature, suture and cure it [22, 89, 94]. If the leakage cannot be identified, fibrin glue is widely used [10, 57, 108–110] such as BioGlue [111], Floseal Hemostatic Matrix topical [94] and so on to cure all potential leakages during surgeris. Peritoneovenous shunts are another way to treat the refractory lymphatic leakage [83, 112]. Since laparoscopic surgeries seem to have a high incidence of lymphatic leakage, it is necessary to coagulate the lymphatic channels adequately as a preventive measure, even if lymphatic leakage is not found [10, 31, 57].

## MATERIALS AND METHODS

### Objective

After searching in PubMed about lymphatic leakage using the words “postoperative” plus “different types of lymphatic leakage” as the subject heading, we couldn't find any article regarding the kinds of iatrogenic lymphatic complications, hence reviews and many diagnoses of lymphatic complications are found confusing due to different definition. The absence of exact definition of postoperative lymphatic leakage makes it difficult to do further analysis and causes more confusion. Thus, this review reveals lymphatic circulation in general, classification and definition of the most of lymphatic leakages, and summarizes the complications, diagnosis and treatment methods. This will provide you an all-sided view of postoperative lymphatic leakage.

## CONCLUSIONS

Even though only 112 closely related articles are referenced, those are enough to show the all-sided postoperative lymphatic leakage, we concluded that the complications, diagnoses, and treatments to provide an all-round view of postoperative lymphatic leakage. The exact definition of different types of postoperative lymphatic leakage benefits us in understanding comprehensively about it and improves our ability to analyze it. Varying incidence and high risk factors will remind us in the prevention of lymphatic leakage.

## SUPPLEMENTARY MATERIALS TABLES







## Abbreviations

ILND: inguinal lymph node dissection
BMI: body mass index
RPLND: retroperitoneal lymph node dissection
POD: post-operation day
MLND: mediastinal lymph node dissection
CT: Computerized tomography
US: ultrasonography
ICG: indocyanine green
MCT: medium-chain triglyceride
TPN: total parenteral nutrition
NPWT: negative pressure wound therapy
TCHM: traditional Chinese herb medicine.
