# Supplementary material for: A review of the postoperative lymphatic leakage
Source: Oncotarget. 2017 Apr 20;8(40):69062–75. doi: 10.18632/oncotarget.17297 (PMC5620321; doi:10.18632/oncotarget.17297)
Supplement: Supplementary file 1 [file oncotarget-08-69062-s001.pdf]

A review of the postoperative lymphatic leakage

Supplementary Materials

Supplementary Table 1: Characters of patients with postoperative lymphatic leakage reported by case reports. See_Supplementary_Table 1

Supplementary Table 2: The characters of patients with postoperative lymphatic leakage reported by incidence. See_Supplementary_Table 2
