# Supplementary material for: A review of the postoperative lymphatic leakage
Source: Oncotarget. 2017 Apr 20;8(40):69062–75. doi: 10.18632/oncotarget.17297 (PMC5620321; doi:10.18632/oncotarget.17297)
Supplement: Supplementary file 2 [file oncotarget-08-69062-s002.doc]

**Supplementary Table 1. Characters of patients with postoperative lymphatic leakage reported by case reports.**

| **Num** | **author** | **Journal** | **types** | **Cases amount** | **age/gender** | **disease** | **operation** | **symptom and complication** | **therapy** | **surgical intervention or not** | **follow-up** | **Diagnosis methods** |
| --- | --- | --- | --- | --- | --- | --- | --- | --- | --- | --- | --- | --- |
| **1** | Glauco Baiocchi [^35^](#_ENREF_35)(2010) | Arch Gynecol Obstet | chylous ascites | 2 | 50y female | serous-papillary ovarian cancer | total abdominal hysterectomy + RPLND | milk whitish fluid(1,470 ml/24 h after oral diet on POD4); nausea; vomiting | drain( POD30); TPN (14d); octreotide(5d); MCT (35d) | No | 10  months | LAB |
|  |  |  |  |  | 63y female | endometrioid adenocarcinoma of the endometrium |  | milk whitish fluid (2,470ml/24h after oral diet on POD3) | drain( until POD14); TPN(10d); octreotide (5d); MCT(21d) |  |  |  |
| 2 | Hashem M.[^36^](#_ENREF_36) (2012) | Eur J Vasc Endovasc | chylous ascites | 1 | 76y male | abdominal aortic aneurysm | conventional aneurysm repair | ascites 1.2-1.5L/d  distending abdomen  decreases appetite  dyspepsia | drain(last 21d when it fell out accidentally); TPN; octreotide; introvenous albumin replacement | YES  peritoneovenous shunt; laporatomy | NA | CT; LAB; lymphangiogram |
| 3 | Daniel W. Suver [^37^](#_ENREF_37)(2004) | Int J Pediatr Otorhi | lymphorrhea; chylous fistula; chylothorax | 1 | 10 months | large cystic mass of neck(11cm×21cm×13cm) | left modiﬁed radical neck dissection; right suprao mohyoid neck dissection; mediastinal dissection via left thoracotomy | drainage(300mL/d) even after reoperation; respiratory (thoracic fluid accumulation); line sepsis; bacteremia | 1.TNP(2m)  2.reoperation  3.MCT(2m)  4.octreotide(7d） | YES | 2 years | NA |
| 4 | J URGEN P  ASSAGE[^38^](#_ENREF_38)(2006) | ANZ J. Surg | lymph leak  chylothorax | 2 | 19y  female | haemangio-lymphoma | thoracic surgical procedure | NA | Drainage; surgure with BioGlue (fail); radiotherapy | YES | NA | LAB |
|  |  |  |  |  | 73y male | advanced lung cancer | lobectomy |  | Drainage; surgure with BioGlue |  |  |  |
| 5 | S. Zeidan[^39^](#_ENREF_39)(2008) | Journal of Pediatric Gastroentreology and Nutrition | chylous ascites | 6 | mean age 4y  4 males  2 females. | abdominal neuroblastoma | radical tumor resection with extensive dissection of the inferior vena cava, abdominal aorta, celiac axis, and superior mesenteric artery. | increasing abdominal girth | TPN(25.3d); duration(all patients); surgery +compressive abdominal garments (3patients); somatostain analogue(1patient) | YES  surgical  absorbable mesh and fibrin glue | 4.3 years  2 patients relapses resolvedspontaneously(1)  MCT(1) | paracentesis+LAB; Technetium-99m; lymphoscintigraphy; lipophilic dye in a concentrated fatty meal |
| 6 | Gopesh K. Modi[^40^](#_ENREF_40) (1999) | Nephrol Dial Transplant | Lymph leakage | 1 | 45y male | end-stage renal failure | right subclavian vein catheter in | serous ooze (3ml/10-15min) 12h after procedure; soaking dressing 10-12 pads/d | prolonged compression, subsequent purse string suture(failed); remove the catheter | NO | NA | NA |
| 7 | Andrea Tinelli[^41^](#_ENREF_41)  (2013) | Int J Gynecol Cancer | lymphocele | 20 | NA | endometrial cancer | type radical hysterectomy; bilateral salpingo-oophorectomy; PLND | pelvic fullness, chills, fever, lower abdominal pain, sciatic compression neuropathy, and palpable pelvic mass. | ultrasound-guided drainage; sclerotherapy with a 0.45% lactic acid solution | NO | NA | US |
| 8 | Qi J[^30^](#_ENREF_30)(2009) | Singapore Med J | Chyloretroperitoneum; retroperitoneal chylous effusion | 2 | 87y  male | clear cell carcinoma of the left kidney | radical resection of the tumour and PALND | milky drainage 300-600mL/d after eating liquid food | Drainage; low-fat diet; anti-acid treatment; somatostain | NO | 19 months | LAB  US |
|  |  |  |  |  | 56y  male | right adrenal phaeochromocytoma | tumor resection | pain of upper back  serous drainage(>200mL/d) | TNP(2w); octreotide(2w) |  | 11  months | percutaneous puncture; CT |
| 9 | Ivo Giovannini[^42^](#_ENREF_42)  (2008) | Nutrition 24 | chyloperitoneum  lymphatic fistula | 2 | 60y male | lymph node metastases | lymphadenectomy | white milky(1300 mL/d POD4) after of oral diet | TNP(25d); octreotide (22d); MCT(28d) | NO | NA | LAB; US |
|  |  |  |  |  | 61y male |  | resection of liver segments with lymphadenectomy | white milky(350 mL/d POD5) after of oral diet | TNP(16d); somatostatin (13d); MCT(28d) |  |  |  |
| 10 | Thilo Hackert[^43^](#_ENREF_43)(2006) | Langenbeck Arch Surg | lymphorrhea lymphatic fistula | 5 | 3 female  2 male | lymph node metastasis or soft tissue tumors | extensive soft tissue resections | After an observation time of 19–27 days, persistent drainage fluid loss occurred in all patients(270-500mL/d) | A single injection 1 g doxycycline via wound or thoracic(23d) | NO | NA | LAB |
| 11 | Ramona Mayer1[^44^](#_ENREF_44)(2005) | StrahlentheR Onkol | lymphocutaneous fistulas  lymphorrhea | 17 | mean age 64y | NA | vena saphena harvesting (7); femoropopliteal bypass (3); surgery for varicose veins (2); hip arthroplasty (3); shunt surgery (1); piercing (1) | NA | radiotherapy(0.3-2 Gy, 7d, range, 2–16 days) | NO | 12.4 months | NA |
| 12 | Philip Touska[^5^](#_ENREF_5)(2012) | BMJ Case Reports | lymphatic leakage  lymphocele  chyle leak | 1 | 58y  female | right-sided multinodular goitre | right thyroid lobectomy | neck swelling at the site of her thyroidectomy; milky drainage (300-400mL/d); wound infection | 1.vaccum drain  2.enteric ultra-low-fat diet  3.octreotide(7d) | NO | NA | LAB |
| 13 | KOJI NISHIZAWA[^10^](#_ENREF_10)  (2006) | Int J Urol | chylous ascites | 1 | 44y female | left renal cancer | laparoscopic transperitoneal left radical nephrectomy with PALND | milky ascites(1000 -4000 mL/d after oral intake on POD3); abdominal fullness; dyspnea; skin rash; moderate leucopenia | 1.Drainage  2.TNP  3.surgery  4.somatosta | laparoscopic lymphostasis; ﬁbrin glue | 3 months | LAB |
| 14 | E. Capocasale[^18^](#_ENREF_18)  (2006) | Transpl P | lymphorrhea  lymphatic leak | 20 | mean age  51y | NA | renal transplantation | povidone group(10 patients):  drainage 164mL/d | draiange(16.3d); iodate povidone solution | NO | NA | LAB |
|  |  |  |  |  |  |  |  | octreotide group(10 patients): drainage 115mL/d | drainage(8.5d); octreotide |  |  |  |
| 15 | HIROMITSU NEGORO[^31^](#_ENREF_31)  (2006) | Int J Urol | chyloretroperitoneum | 2 | 71y  male | carcinoma of the renal pelvis | retroperitoneal laparoscopic left nephroureterectomy | continuous pain on his low back and left low abdomen; drainage 1000ml/d | Drainage; fat restricted diet; TNP; octreotide | NA | 5 months | CT |
|  |  |  |  |  | 68y  male | carcinoma of the lower ureter | retroperitoneal laparoscopic left nephroureterectomy and open partial cystectomy | drainage(220-400 mL/d, turning to chylous-like ﬂuid after feeding on POD2) | 1.drainage  2. sclerotherapy with povidone iodine  3.octreotide |  |  |  |
