# Supplementary material for: A review of the postoperative lymphatic leakage
Source: Oncotarget. 2017 Apr 20;8(40):69062–75. doi: 10.18632/oncotarget.17297 (PMC5620321; doi:10.18632/oncotarget.17297)
Supplement: Supplementary file 3 [file oncotarget-08-69062-s003.doc]

**Supplementary Table 1. The characters of patients with postoperative lymphatic leakage reported by incidence.**

| **Num** | **author** | **journal** | **types** | **morbidity** | **age/gender** | **disease** | **operation** | **symptom and complication** | **therapy** | **surgical intervention or not** | **follow-up** | **diagnosis** |
| --- | --- | --- | --- | --- | --- | --- | --- | --- | --- | --- | --- | --- |
| 1 | Wei Jiang[^45^](#_ENREF_45)(2012) | Surg Laparosc Endosc Percutan Tech | ascites | 8/ 21,380 | 23-54y | uterine myomas (2); ovarian endometrioid cyst(2); tubal pregnancy (2); adenomyoma(1); ovarian mature teratoma (1) | laparoscopic surgeries | ascites (1,650-9,200mL) on POD1-5; abdominal distention; nausea; vomiting | supportive treatments ; prophylactic antibiotics | NO | 1 month | LAB; US |
| 2 | Choon seow[^6^](#_ENREF_6)(2010) | international journal of surgery | lymph leak;  chylous ascites | 36/442 | Median age 62y 14female 22male | NA | Whipple’s procedure(13); oesophagectomy(10); abdominal aneurysm repair(1); pulmonary resection(1); radical adrenalectomy(1) | drainage(>500mL over 48h); ALOS 39d | 67% TPN alone (16 d); 33% surgery procedures | (33%): peritoneovenous shunt; re-intervention thoracic surgery | NA | NA |
| 3 | Jeanwan L.[^46^](#_ENREF_46)(2008) | Journal of vascular surgery | lymph leak | 1/58 | NA | occlusive disease | common femoral endarterectomy (CFE) | NA | all self-limited and treated in an outpatient setting | NO | 1year | NA |
| 4 | Michael Milonakis[^9^](#_ENREF_9)(2009) | J Card Surg | chylothorax | 1 8 /1,341 | median 19.5 months; 9 males; 9 females | congenital heart disease | congenital heart surgery | drainage milk fluid (5.6 mL/kg per day) | TNP(all patients); somatostain analogue (6 patients); MCT(all patients, 6weeks) | surgical intervention(3 patients) | NA | LAB |
| 5 | Kerstin Lorenz[^22^](#_ENREF_22)  (2010) | Langenbecks Arch Surg | lymph fistula; chyle fistula; chylous leakage | 29/5,736 | 17males  12females | benign nodular goiter (2); recurrent goiter (1); thyroid carcinoma (10); thyroid carcinoma recurrence (16). | thyroid surgery | drainage (198-322 ml/day); wound infection (1); ALOS (11d conservative group ;21d surgical group) | drainage(all); TNP(all); pressure dressing(all); surgical procedures (10 patients) | ligation, suture,  muscle flap  fibrin glue | recurrences(7); conservative group (5); surgical group (2) | LAB |
| 6 | M. Cnotliwy[^47^](#_ENREF_47)  (2001) | Eur J Vasc Endovasc Surg | lymphocutaneous ﬁstula | 19/692 | median age 66y 14 males 5 females | NA | primary lower limb arterial reconstructive procedures | clear, transparent, sterile fluid | group 1, 11 patients (healing time 3d): doxycycline hydrochloride solution | NO | 3 months | NA |
|  |  |  |  |  |  |  |  |  | group 2, 8 patients (healing time 17d):compressive dressing; prophylactic antibiotic therapy with cefradine |  |  |  |
| 7 | Pengfei Shao[^48^](#_ENREF_48)(2010) | BJU International | lymphatic leak; lymphatic leakage | 17/43 | NA | bladder carcinoma | laparoscopic radical cystectomy with extended PLND | clear pelvic lymphatic drain(100–600 mL/d) | drainage 2-3w (heal itself without further intervention) | NO | 3 months | CT |

Table1&2:

Abbreviation: NA: Not available; PRLND: pelvic and retroperitoneal lymph node dissection; PLND: pelvic lymph node dissection; PALND: para-aortic lymph nodes dissection; US: ultrasonography; LAB: Laboratory examination; POD: postoperative day; TPN: total parenteral nutrition; MCT: dietary intervention-medium-chain triglycerides; ALOS: average length of stay.

Notes: POD X: the symptom started from the X day after the operation; POD X d/w: the therapy last X days or X weeks; (X) in therapy column: this kind of therapeutic method was used in X patients.
